# Is model-based dose calculation based on cone-beam computed tomography suitable for adaptive treatment planning in brachytherapy?

**DOI:** 10.1007/s00066-024-02318-3

**Published:** 2024-11-27

**Authors:** Andre Karius, Maya Shariff, Sabrina Schaller, Michael Lotter, Vratislav Strnad, Niklas Lackner, Rainer Fietkau, Christoph Bert, Ricarda Merten, Claudia Schweizer

**Affiliations:** 1 Department of Radiation Oncology, Universitätsklinikum Erlangen, Friedrich-Alexander-Universität Erlangen-Nürnberg (FAU), Universitätsstraße 27, 91054 Erlangen, Germany; 2 Comprehensive Cancer Center Erlangen-EMN (CCC ER-EMN), Erlangen, Germany

**Keywords:** Model-based dose calculation, Brachytherapy, Breast cancer, Cervical cancer, Adaptive radiotherapy

## Abstract

**Background and purpose:**

Model-based dose calculation considering tissue compositions is increasingly being investigated in brachytherapy. The aim of this study was to assess the suitability of modern cone-beam computed tomography (CBCT) imaging compared to conventional computed tomography (CT) scans for this purpose.

**Materials and methods:**

By means of a phantom study, we evaluated the CT numbers and electron densities measured using a modern CBCT device as well as a conventional CT scanner for various materials. Based on this, we compared dose calculations (using the TG-43 formalism as well as model-based collapsed cone calculations assuming uniform materials [ACE_uniform_] and considering CT numbers [ACE_CT#_]) on planning CTs and control CBCTs for patients with cervical and breast cancer as well as phantom-simulated skin cancer cases. Assessing dosimetric deviations between the planning CTs and control CBCTs acquired during the treatment course served to estimate interfractional implant variations.

**Results:**

The comparison of ACE_uniform_–ACE_CT#_ deviations between planning CTs and control CBCTs revealed no statistically significant difference for almost all examined dose parameters. Dosimetric deviations between model-based dose calculations and TG-43 were partly significant but of small magnitude (< 10 cGy per fraction). Interfractional dosimetric variations were substantially larger than the dosimetric differences found between the various dose calculation procedures.

**Conclusion:**

Model-based dose calculation based on modern CBCT imaging was suitable. However, the found differences between these calculations and the TG-43 formalism should be investigated in dose–outcome analyses. The observed interfractional dosimetric variations revealed the importance of performing treatment quality assurance.

## Introduction

Adaptive workflows have gained rising importance for brachytherapy of several entities, as for instance cervical cancer [1, 2], prostate cancer [3–5], and breast cancer [6–8]. For example, for increasing the accuracy of applicator insertions with respect to the underlying patient case, supporting tools like pre- and intraoperative imaging modalities [9–13], tracking systems [14–16], or even robots [17, 18] are increasingly being tested and utilized. Moreover, identifying the need for treatment adaption (e.g., by treatment replanning [19, 20]) due to dosimetric variations potentially occurring during the brachytherapy course is considered beneficial to improve the therapy of affected patients.

Considering the treatment adaption to specific clinical requirements, an extended focus should also be put on the brachytherapy dose calculation. The latter is nowadays mostly performed using the TG-43 formalism, i.e., assuming a water environment with full scatter conditions around the radiation source [21]. Anatomical information gained from, e.g., computed tomography (CT) scans appear during treatment planning only as image background, without any effect on the resulting dosimetry. However, at bone–tissue or air–tissue transitions, the dose rate actually delivered can be significantly altered up to 11.5% at 5 cm distance from the source [22, 23], due to associated variations in absorption and backscatter. Dosimetric benefits resulting from image-based adaptive brachytherapy workflows might be reduced if the dosimetry itself is affected by formalism-related uncertainties or miscalculations. The usage of so-called model-based dose calculation algorithms (MBDCAs), which take into account material/tissue heterogeneities and are described in the Report of the Task Group 186 (TG-186) of the American Association of Physicists in Medicine (AAPM) [24], is therefore aimed at improving dose calculation accuracy.

For the application of MBDCAs, establishing a correct assignment between Hounsfield units of planning CT scans and the density of considered tissues (e.g., soft tissue, bone, lung) is decisive. Since their introduction, MBDCAs have been limited to the utilization of conventional CT scanners, since cone-beam CT (CBCT) usually revealed a substantially worse CT number accuracy in comparison [25–27]. The TG-186 recommendation even explicitly states that “CBCT images should not be used for brachytherapy dose calculations when tissue heterogeneity information needs to be taken into account” [24]. However, CBCT devices offer partly a high flexibility for mobile imaging in various rooms of a brachytherapy ward [10, 28]. The possibility to use MBDCAs based on CBCT images as well would therefore allow to create holistic on-site workflows. In this debate, it has to be noted that more than a decade of technical advancements has passed since publication of the TG-186 recommendation, and novel CBCT devices launched for various applications already provided high CT number accuracy in phantom studies [28, 29].

Considering these aspects, the aim of this work was to assess the dosimetric deviations resulting from model-based dose calculation based on modern control CBCTs compared to respective calculations on planning CTs for cervical, breast, and simulated skin cancer cases. These clinical scenarios were selected to investigate the validity of the aforementioned recommendation’s statement both deep within the body as well as for target volumes closer to or even at the surface. In addition, we evaluated the CT number accuracy of the used CBCT and CT system, and analyzed for each clinical scenario the dosimetric variations occurring during the brachytherapy course.

## Materials and methods

### Advanced Collapsed Cone Engine dose calculation

In the present work, we applied the Advanced Collapsed Cone Engine (ACE) [30, 31] integrated into the treatment planning system (TPS) Oncentra Brachy (version 4.6.2; Nucletron, Veenendaal, The Netherlands) as MBDCA. Corresponding dose calculations require knowledge about the tissue types and densities present within the anatomical region of interest (ROI). All respective organs and tissues (e.g., bone, lung, air, general soft tissue) therefore have to be contoured on an acquired CT scan and can subsequently be linked to one of the tissues included in a material table predefined by the TPS distributor, which in general refers to Table III of the TG-186 recommendation [24]. Dose calculation can then be performed assuming for each assigned material a uniform density, which is also taken from the aforementioned Table III. This way of dose calculation will be referred to as ACE_uniform_ in the following.

As an alternative, the attenuation characteristics of the materials included in each CT voxel can be considered for dose calculation beyond the assumption of uniformity. For this purpose, the respective CT numbers have to be converted to electron densities as commonly performed for external beam radiation therapy (EBRT). In the used TPS, this conversion is conducted according to Knöös et al. [32]:

1 $$\begin{array}{l}\rho _{e}=\left(A+\frac{B\cdot CT_{\textit{number}}}{1000}\right).10^{23}\frac{1}{cm^{3}},\\[+2mm]\text{with}\,\begin{cases} A=3.30\,\text{and}\,B=\frac{3.40}{HU}\,\text{for}\,CT_{\textit{number}}\leq 150\,\mathrm{HU}\\ A=3.65\,\text{and}\,B=\frac{1.22}{HU}\,\text{for}\,CT_{\textit{number}}> 150\,\mathrm{HU} \end{cases} \end{array}$$

*ρ*_*e*_ and *CT*_*number*_ refer to the electron density and the voxel’s CT number in Hounsfield units, respectively. *A* and *B* represent conversion parameters. The electron density obtained in this way is then directly related to the mass density of the examined tissue, following International Commission on Radiation Units and Measurements (ICRU) reports 44 [33] and 46 [34]. Based on this information, dose calculations considering the attenuation characteristics of each voxel can be performed, which will be referred to as ACE_CT#_ in the following.

Both calculations ACE_uniform_ and ACE_CT#_ were performed using the Advanced Collapsed Cone Engine (ACE) integrated into our TPS as mentioned above. The description of the underlying algorithm was provided in detail earlier [30, 31, 35–38] and shall not be repeated here. In brief, it calculates the total dose to the local medium transported through the medium as sum of the dose deposited by primary photons (obtained by a ray tracing procedure) and the dose resulting from single and multiple scattered photons (obtained via a collapsed cone superposition convolution method). Based on this approach, we investigated the suitability of model-based dose calculation on CBCT scans as follows.

### CT scanners and CT number phantom examinations

Following Eq. 1, a high CT number accuracy is important for the intended model-based dose calculations. In this work, we analyzed the feasibility of achieving this requirement with modern state-of-the-art CBCT compared to conventional CT. As CBCT device, the mobile 3‑in‑1 X‑ray system ImagingRing m (medPhoton, Salzburg, Austria) characterized in detail previously [28, 29] was utilized. Due to its mobility, the device is well-suited for on-site imaging on the brachytherapy ward and was used to acquire control CBCT scans as outlined in section ‘Patient workflow and anthropomorphic phantom study’. For conventional CT imaging, a SOMATOM go.Open Pro scanner (Siemens, Forchheim, Germany) referring to our default system for the acquisition of planning CTs was applied.

**Fig. 1:**
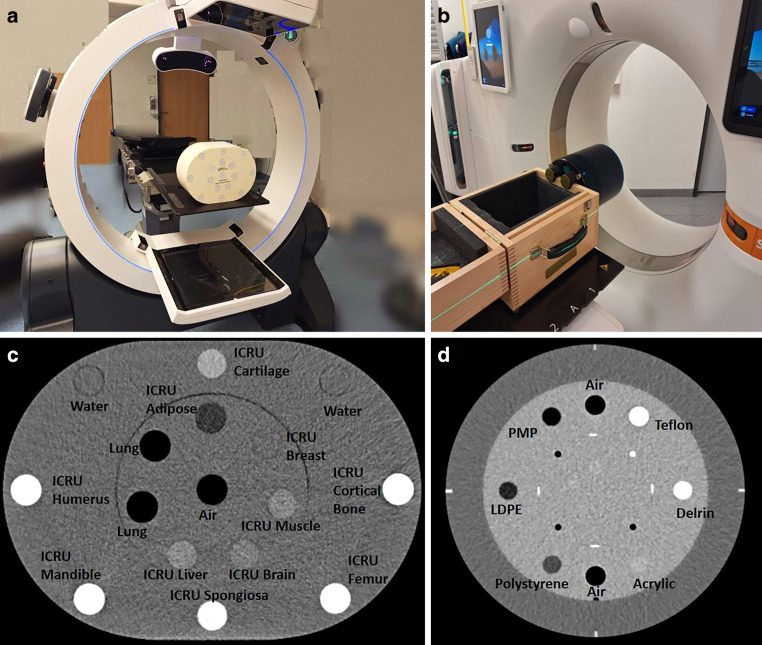
CBCT scanner ImagingRing m (**a**) and the conventional CT scanner Somatom go.Open Pro (**b**), with the examined phantoms being exemplarily placed within the gantry. The inserts contained in the Comprehensive Electron Density Phantom (**c**) and the CatPhan 504 (**d**) are labeled in each case and represented a broad human body like CT number spectrum. *ICRU* International Commission on Radiation Units and Measurements

To evaluate CT number accuracy for both machines, phantom studies considering the CatPhan 504 (The Phantom Laboratory, Salem, USA) as well as the Comprehensive Electron Density Phantom (QRM, Möhrendorf, Germany) were conducted first. Both phantoms contain inserts of various materials with different electron densities (provided by the manufacturers) that represented the broad CT number spectrum of a typical human body (Fig. 1). The CatPhan was used since it is a widespread standard quality assurance (QA) phantom in radiotherapy, but comprises only materials with an effective atomic number of approximately seven [39], meaning that bony tissue is not sufficiently represented. To account for this drawback, the Comprehensive Electron Density Phantom was also examined. This is specifically designed [40] to mimic human tissue (including bony structures) regarding electron density according to ICRU report 44 [33] and 46 [34].

As illustrated in Fig. 1, the two phantoms were placed isocentrically in the CT and CBCT devices and scanned five times each. Scan parameters of the SOMATOM go.Open Pro CT scanner included 120 kV tube voltage, automatic tube–current modulation, continuous radiation output, 0.5 s gantry rotation time, 0.4 × 0.4 × 2 mm^3^ voxel size, 64 × 0.6 mm collimation, smooth Br40 kernel, and spiral-scanning mode. Scan parameters of the ImagingRing CBCT included 120 kV tube voltage, automatic tube–current modulation, pulsed radiation output with 30 Hz frame rate, 20 s gantry rotation time, 0.4 × 0.4 × 2 mm^3^ voxel size, 300 μm detector pixel pitch, Shepp–Logan kernel, and circular-scanning mode. No user-selectable artifact reductions were applied for both the CT and CBCT system. It should be noted that based on previous reports [28, 29], the ImagingRing features only heuristic scatter corrections. On the central slice of the acquired images, a circular ROI (10 and 18 mm diameter for the CatPhan and the Comprehensive Electron Density Phantom, respectively, which was about 2 mm smaller than the inserts’ diameter to reduce the impact of CT number fluctuations at their edges) was placed centered within each insert by using an adapted version of the software QAMaster [41] to determine the respective mean CT number. CT numbers and associated electron densities (resulting from Eq. 1) were then compared between the two scanners to estimate the CT number inaccuracies occurring with modern CBCT imaging.

### Patient workflow and anthropomorphic phantom study

To evaluate the impact of CT number uncertainties on model-based dose calculation, we examined 18 cervical and 8 breast cancer patients as well as an anthropomorphic upper torso phantom (Radiology Support Devices, Gardena, CA, USA). Our clinical workflows and the phantom setup are described in the following. All dose calculations performed during treatment planning were based on TG-43 as well as considering an ^192^Ir radiation source (microSelectron mHDR-v2r source for pulsed dose rate (PDR) treatments; Flexisource 192-Ir source for high dose rate (HDR) treatments; both provided by Nucletron). All image acquisitions were performed as part of our default workflow. No IRB approval or similar was required for the present work.

**Fig. 2:**
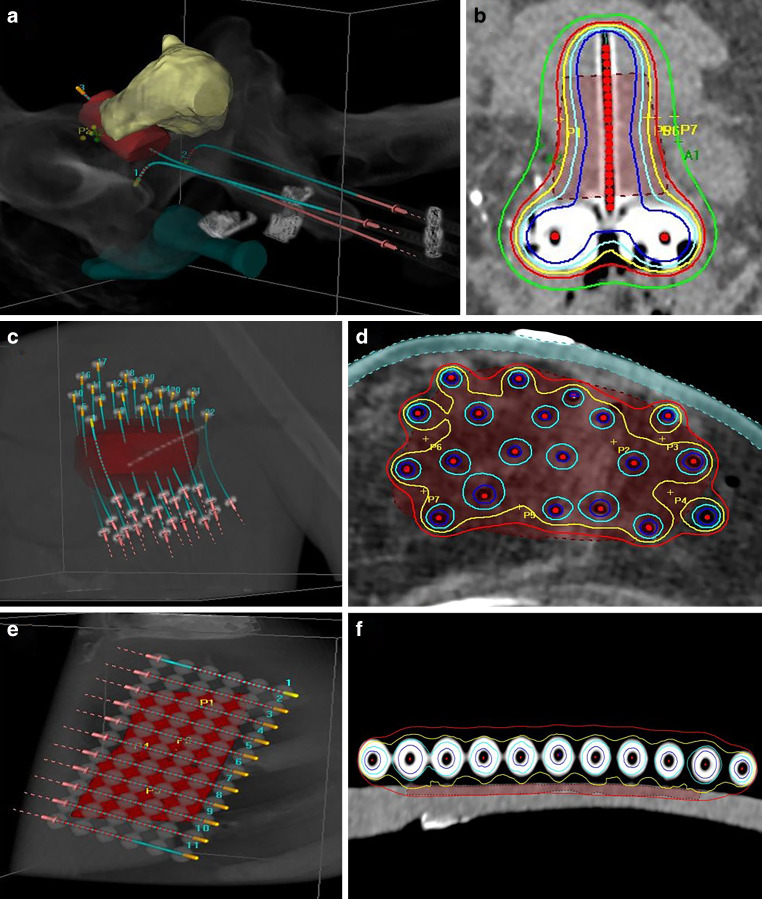
Three-dimensional representations of a typical cervix (**a**), breast (**c**), and phantom surface (**e**) case with reconstructed applicators (blue) and target volumes (red) to provide an impression of the dose distributions examined in the present work. For the cervix case, bladder and rectum are additionally shown in yellow and green, respectively. The dose distributions planned for the cervix (**b**), breast (**d**), and phantom (**f**) case are shown as well. Here, the red, yellow, cyan, blue, and green lines refer to the 100, 120, 150, 200, and 70% isodose curves, respectively. The corresponding target volume is shown as filled red contour

The considered cervical cancer patients received PDR brachytherapy with single doses of 0.6 Gy every hour for 24 h per day up to a total dose of 40–45 Gy as boost following EBRT. For this purpose, a Fletcher applicator was implanted based on intraoperative ultrasound-guidance, accompanied by an interstitial insertion of additional plastic needles in 10 of the 18 cases. Afterwards, high-risk clinical target volume (HR-CTV) as well as organs at risk (OARs; bladder, rectum) were contoured on an acquired planning CT following GEC-ESTRO guidelines [42]. Treatment planning aimed to achieve a HR-CTV D_90_ (dose the most exposed 90% of the structure volume receive) of ≥ 100% of the prescribed dose, while keeping $\mathrm{D}_{{2\mathrm{cm}^{3}}}$ (EQD2) of bladder and rectum below 85 and 75 Gy, respectively (including EBRT exposure). A typical case and planned dose distribution is shown in Fig. 2a, b. At about halfway through the brachytherapy course, a control CBCT was acquired to assess potential geometric implant variations as described previously [10].

The examined breast patients received HDR multicatheter brachytherapy considering the recently reported workflow [8] and a fractionation of 9 × 3.8 Gy. The interstitial insertion of plastic catheters into the breast (which were fixed with plastic buttons at the skin to avoid slippage) was followed by a planning CT acquisition, on which planning target volume (PTV) and OARs (ribs, skin) were delineated according to GEC-ESTRO guidelines [43]. Treatment planning aimed to achieve a PTV D_90_ ≥ 100% of the prescribed dose. Ribs $\mathrm{D}_{{1\mathrm{cm}^{3}}}$ and $\mathrm{D}_{{0.1\mathrm{cm}^{3}}}$ as well as skin $\mathrm{D}_{{1\mathrm{cm}^{3}}}$ and $\mathrm{D}_{{0.2\mathrm{cm}^{3}}}$ should receive < 80% and < 90% as well as < 90% and < 100% of the prescribed dose, respectively. A typical patient case is exemplarily shown in Fig. 2c, d. After the fourth irradiation fraction, a control CBCT was acquired to detect implant variations as described previously [8].

Furthermore, since we currently acquire no control CBCTs of our patients suffering from superficial tumors, the aforementioned upper torso phantom was utilized. The phantom simulates a human thorax and comprises, next to Rando plastic simulating soft tissue, particularly artificial ribs and lung. On top of the phantom chest (Fig. 2e, f), we fixed in succession six different Freiburg flaps (Elekta, Veenendaal, The Netherlands) of sizes 5 × 10 cm^2^ to 9 × 20 cm^2^ using tape to avoid slippage. A planning CT was acquired in each case to contour a 1–2 mm deep PTV with the planar dimension of the respective flap right underneath this flap. No OARs were considered following our clinical workflow of treating corresponding patients. Afterwards, a treatment plan with PTV D_90_ ≥ 100% for a PDR treatment with 100 pulses of 0.5 Gy (irradiated every hour) up to a total dose of 50 Gy was created. Moreover, we acquired for each fixed flap a corresponding CBCT scan to assess CBCT-based dose calculations for these superficial treatments as well.

### Analysis of dosimetric implant alterations

To analyze dosimetric variations during the treatment course for each entity and patient, the respective OARs were recontoured on the control CBCT and the target volume was transferred to the latter from the planning CT. For cervix patients, this was based on a rigid registration of the Fletcher applicator within Oncentra Brachy. For breast patients, the target volume transfer was performed by means of a deformable image registration within RayStation (RaySearch, Stockholm, Sweden) [6], after which the image and associated structure data sets were reimported in Oncentra Brachy for dose calculations. In each case, the goodness of these transfer procedures was evaluated by assigning the registration quality score described in the AAPM TG-132 report [44].

Subsequently, the treatment plan (i.e., the dwell positions and times of the source) was transferred to the applicator arrangement obtained on the control CBCT. The resulting dose distribution was then compared to the initial treatment plan considering the dose metrics mentioned in the section ‘Patient workflow and anthropomorphic phantom study’ as well as the volumes V_100_ and V_150_ enclosed by the 100 and 150% isodose surfaces, respectively. These clinical assessments and comparisons were conducted solely based on TG-43 dose calculations.

For the anthropomorphic phantom, no corresponding evaluation was performed due to the absence of implant variations in this case and in order to avoid a misinterpretation of the results with respect to clinical patients. Nevertheless, the respective target volume was transferred from the planning CT to the control CBCT by means of a rigid registration of the phantom surface within Oncentra Brachy for the following investigations (section ‘Comparison of dose calculation methods’). Registration quality was assessed as above.

### Comparison of dose calculation methods

As final step of the present work, to assess the suitability of Advanced Collapsed Cone Engine dose calculations based on CBCT images, we recalculated the TG-43 dose distributions created on both planning CTs and control CBCTs by means of ACE_uniform_ and ACE_CT#_. All ACE calculations were performed with the standard resolution setting of Oncentra Brachy being selected. For this purpose, following tissues/materials listed in Table III of the TG-186 recommendation [24] were manually contoured on the CTs and CBCTs as required according to section ‘Advanced collapsed cone engine dose calculation’:

- For cervix cases: bones, air, and general female soft tissue;
- For breast patients: bones, skin, air, lung, and general female soft tissue; and
- For the phantom cases: bones, air, lung, and general female soft tissue.

All three dose distributions (TG-43, ACE_uniform_, ACE_CT#_) were compared to each other on both planning CTs and control CBCTs considering the dose metrics mentioned above. In this respect, the comparison of TG-43 to ACE_uniform_ enabled a quantification of the dosimetric impact of the varied dose calculation algorithm itself. The dosimetric effects of explicitly considering the CT numbers of each voxel were assessed by comparing ACE_uniform_ to ACE_CT#_. Statistical significance of found deviations was tested using a Wilcoxon signed-rank tests at 5% significance level. The comparison of the ACE_uniform_–ACE_CT#_ variations between planning CT and control CBCT served for evaluating the suitability of CT-number-based dose calculations on CBCT images compared to conventional CT. The differences in these variations were tested for statistical significance by means of Mann–Whitney U tests at the 5% significance level.

## Results

### CT number phantom examinations

**Fig. 3:**
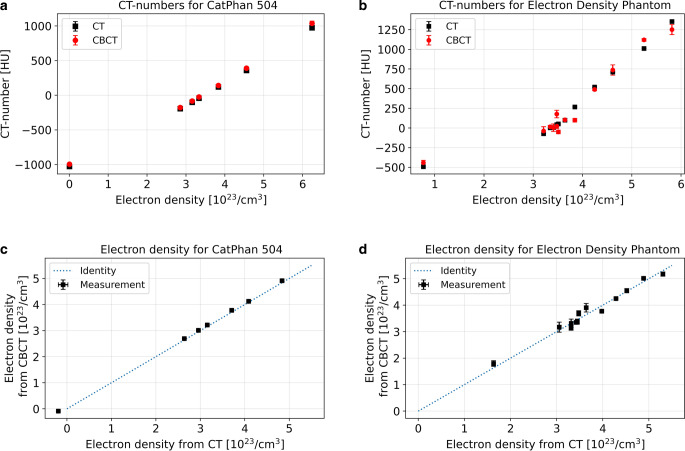
CT numbers obtained for the CatPhan (**a**) and Comprehensive Electron Density Phantom (**b**) with the CT and CBCT device. The electron densities of the various inserts as specified by the phantom manufacturers are indicated on the abscissa of these graphs. Furthermore, the electron densities calculated from Eq. 1 for both CT and CBCT scans are plotted against each other for the CatPhan (**c**) and Comprehensive Electron Density Phantom (**d**) to visualize the compliance of the CBCT results with the CT. The uncertainty bars represent the standard deviation of the results considering all five conducted measurements in each case. For better comparability, the identity function is illustrated as well

The CT numbers obtained from the CT and CBCT scans of both examined phantoms are shown in Fig. 3. For the CatPhan, a mean CT number difference between CT and CBCT of −29 ± 17 HU (range [−64 HU, −16 HU]) was obtained considering all inserts, which resulted in deviations of the calculated electron densities of (−6.8 ± 2.3) × 10^21^/cm^3^ (range [−1.1 × 10^22^/cm^3^, 4.0 × 10^21^/cm^3^]). These were one to two orders of magnitude smaller than the electron densities specified for the individual insert materials (except of air) as indicated on the abscissae in Fig. 3a, b.

For the Comprehensive Electron Density Phantom, we observed larger CT number deviations between CT and CBCT of up to 167 HU for spongiosa and −135 HU for muscle. The mean deviation considering all inserts amounted 3 ± 84 HU, which resulted in electron density differences of (−1.6 ± 19.3) × 10^21^/cm^3^ (range [−4.6 × 10^22^/cm^3^; 3.5 × 10^22^/cm^3^]). Although these deviations were larger than obtained for the CatPhan, they were again one to two orders of magnitude smaller than the specified electron densities of the individual inserts. In particular, no dependence of the CT numbers on the exact placement of the inserts within the phantom was found. As visualized by plotting the results obtained for both scanners and phantoms against each other (Fig. 3c, d), a reasonable agreement following the identity function was found between CT and CBCT in each case. The transfer of these findings to clinical treatment plans was assessed below.

### Dosimetric implant alterations

**Fig. 4:**
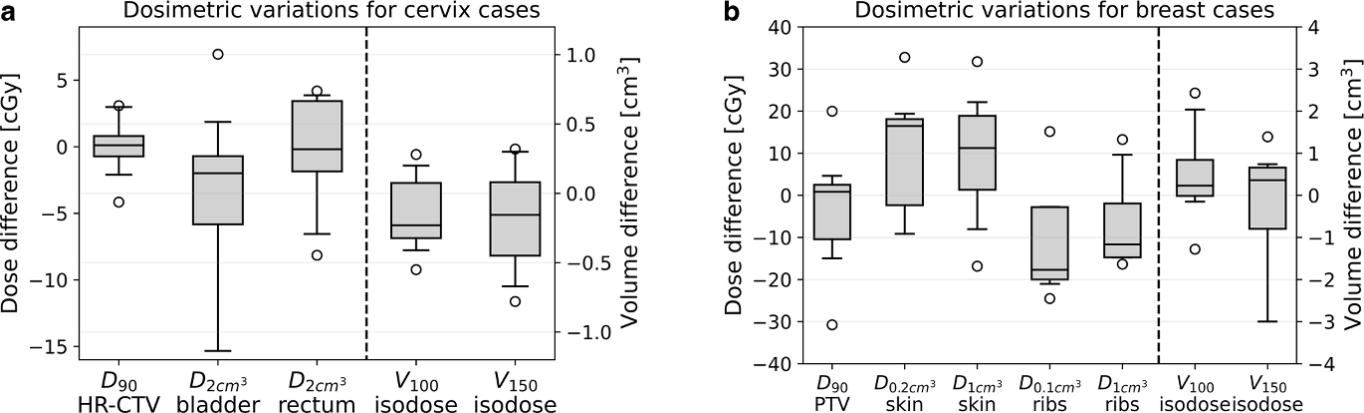
Dosimetric deviations (for single fractions) observed between planning CT and control CBCT situation for cervix (**a**) and breast (**b**) patients considering the various examined dose parameters for target volume and organs at risk. Dose calculations were performed using the TG-43 formalism. In the boxplots, horizontal lines indicate the median, boxes refer to the interquartile range, and whiskers include the 95th percentile of the results, respectively. Outliers are illustrated as circles

Implant variations during the treatment course were evaluated for cervix and breast patients based on the acquired planning CT and control CBCT scans and considering the TG-43 formalism for dose calculations. The results obtained for all examined dose metrics are provided in Fig. 4 and Table 1. All performed image registrations reached a registration quality score according to the AAPM-TG 132 report [44] of ≤ 1 and were thus of high quality.

**Table 1:** Dose–volume parameters (mean ± standard deviation) investigated for the various entities. Dose calculations were performed using the TG43-formalism as well as the model-based dose calculations ACE_uniform_ and ACE_CT#_ on both planning CTs and control CBCTs. Reporting of the results is limited to significant numbers for clarity

Parameter	Planning CT			Control CBCT		
	TG-43	ACE_uniform_	ACE_CT#_	TG-43	ACE_uniform_	ACE_CT#_
*Cervix*						
D_90_ HR-CTV [cGy]	65 ± 6	65 ± 6	65 ± 6	65 ± 7	65 ± 7	65 ± 7
$\mathrm{D}_{{2\mathrm{cm}^{3}}}$ bladder [cGy]	39 ± 12	39 ± 12	39 ± 12	44 ± 11	44 ± 11	44 ± 11
$\mathrm{D}_{{2\mathrm{cm}^{3}}}$ rectum [cGy]	24 ± 9	24 ± 9	23 ± 9	24 ± 7	23 ± 7	23 ± 7
V_100_ [cm^3^]	61 ± 23	61 ± 23	61 ± 23	61 ± 23	61 ± 23	61 ± 23
V_150_ [cm^3^]	31 ± 12	31 ± 12	31 ± 13	31 ± 12	31 ± 13	31 ± 13
*Breast*						
D_90_ PTV [cGy]	403 ± 17	402 ± 17	402 ± 17	407 ± 20	405 ± 20	405 ± 20
$\mathrm{D}_{{0.1\mathrm{cm}^{3}}}$ ribs [cGy]	231 ± 78	230 ± 77	228 ± 77	243 ± 89	239 ± 86	237 ± 86
$\mathrm{D}_{{1\mathrm{cm}^{3}}}$ ribs [cGy]	194 ± 61	192 ± 59	190 ± 59	201 ± 67	196 ± 65	195 ± 65
$\mathrm{D}_{{0.2\mathrm{cm}^{3}}}$ skin [cGy]	333 ± 22	328 ± 22	328 ± 22	331 ± 52	324 ± 49	325 ± 49
$\mathrm{D}_{{1\mathrm{cm}^{3}}}$ skin [cGy]	301 ± 8	296 ± 8	296 ± 8	292 ± 18	284 ± 17	285 ± 17
V_100_ [cm^3^]	86 ± 45	85 ± 45	85 ± 45	85 ± 46	85 ± 45	85 ± 46
V_150_ [cm^3^]	22 ± 11	21 ± 11	21 ± 11	22 ± 13	22 ± 13	22 ± 13
*Phantom*						
D_90_ PTV [cGy]	52.4 ± 0.6	51.7 ± 0.9	51.9 ± 0.8	49 ± 6	49 ± 7	49 ± 7
V_100_ [cm^3^]	94 ± 30	84 ± 27	83 ± 27	90 ± 30	83 ± 28	82 ± 27
V_150_ [cm^3^]	39 ± 10	31 ± 8	30 ± 8	36 ± 12	34 ± 10	33 ± 10

With respect to cervix cancer patients, we found a good dose stability regarding the HR-CTV, with D_90_ variations of 0.1 ± 1.8 cGy per fraction or 0.0 ± 1.1 Gy for the entire treatment course, respectively. The maximum D_90_ variation for an individual patient amounted to −3 Gy for the entire course. While the volumes V_100_ and V_150_ enclosed by the 100 and 150% isodose surfaces revealed only small variations with an average of −0.1 ± 0.2 cm^3^ and −0.2 ± 0.3 cm^3^, respectively, larger changes were found for rectum and bladder. For the bladder (mean deviation: −5 ± 13 cGy per fraction), the maximum $\mathrm{D}_{{2\mathrm{cm}^{3}}}$ decrease and increase between planning CT and control CBCT situation were 7 cGy and 15 cGy per irradiation, referring to 6 and 9 Gy for the entire treatment course (assuming an identical anatomical situation for each fraction). For the rectum, $\mathrm{D}_{{2\mathrm{cm}^{3}}}$ changes ranged from −8 cGy to 4 cGy for single fractions and from −5 to 3 Gy for the complete brachytherapy.

For breast patients, all dosimetric deviations reported in the following refer only to the fourth of all nine 3.8 Gy fractions, when control CBCTs were acquired. In this respect, we observed very small PTV D_90_ variations of −3 ± 15 cGy (range [−31 cGy; 20 cGy]) as well as reasonable isodose volume changes of 0.4 ± 1.1 cm^3^ (V_100_) and −0.6 ± 2.2 cm^3^ (V_150_), respectively. Regarding skin exposure, we found $\mathrm{D}_{{0.2\mathrm{cm}^{3}}}$ and $\mathrm{D}_{{1\mathrm{cm}^{3}}}$ deviations between planning CT and control CBCT situation of 1 ± 30 cGy (range [−68 cGy; 33 cGy]) and 8 ± 15 cGy (range [−17 cGy; 32 cGy]), respectively. For the ribs, $\mathrm{D}_{{0.1\mathrm{cm}^{3}}}$ and $\mathrm{D}_{{1\mathrm{cm}^{3}}}$ deviations of −11 ± 13 cGy (range [−25 cGy; 15 cGy]) and −6 ± 11 cGy (range [−16 cGy; 13 cGy]) were observed. Assuming the control CBCT situation to hold for the entire treatment course, one examined patient would have shown a skin $\mathrm{D}_{{0.2\mathrm{cm}^{3}}}$ of 4.4 Gy and one additional patient a ribs $\mathrm{D}_{{0.1\mathrm{cm}^{3}}}$ of 3.4 Gy, meaning that the corresponding GEC-ESTRO implant quality criteria [43] of a skin $\mathrm{D}_{{0.2\mathrm{cm}^{3}}}$ of < 100% and a ribs $\mathrm{D}_{{0.1\mathrm{cm}^{3}}}$ < 90% of the prescribed dose, respectively, were missed. For both breast and cervix patients, the OARs were thus comparatively more affected by dosimetric variations during the treatment course than the actual target volume.

### Comparison of dose calculation methods

To analyze the impact of considering materials and CT numbers for dose calculation, the doses obtained using TG-43, ACE_uniform_ and ACE_CT#_ based on both planning CTs and control CBCTs were compared to each other. Individual cancer entity specific results are summarized in Table 1.

**Fig. 5:**
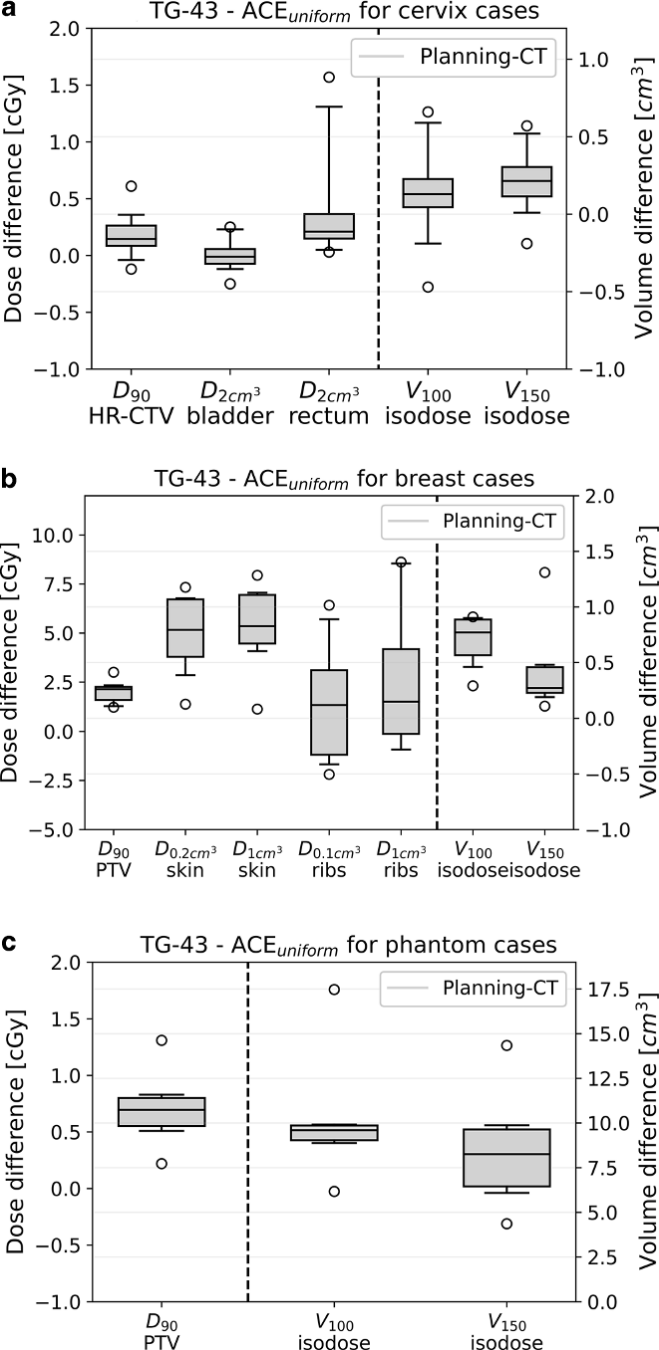
Dosimetric deviations (for single fractions) between TG-43 and ACE_uniform_ obtained exemplarily on the planning CTs for the examined cervix (**a**), breast (**b**), and phantom (**c**) cases. Structure-related differences were always in the cGy range and, thus, appeared to be of only limited clinical relevance

For evaluating the dosimetric effects of model-based dose calculations, the results obtained using the TG-43 formalism were at first compared to ACE_uniform_ calculations considering uniform materials. The differences between both procedures are provided exemplarily for the planning CT in Fig. 5. A corresponding statistical significance between TG-43 and ACE_uniform_ was found for almost all examined structures (*p* ≤ 0.03), with the exceptions of the $\mathrm{D}_{{2\mathrm{cm}^{3}}}$ bladder (*p* = 0.83) for cervix cases as well as V_100_ (*p* = 0.20), ribs $\mathrm{D}_{{0.1\mathrm{cm}^{3}}}$ (*p* = 0.31), and ribs $\mathrm{D}_{{1\mathrm{cm}^{3}}}$ (*p* = 0.15) for breast cases. Maximum dosimetric differences in this respect were found for breast cases for skin $\mathrm{D}_{{1\mathrm{cm}^{3}}}$, which received a mean exposure of 301 ± 8 cGy (TG-43) and 296 ± 8 cGy (ACE_uniform_) on the planning CT. Despite their statistical significance, the observed deviations of TG-43 and ACE_uniform_ appeared to be of only limited clinical relevance. An exception were the results obtained for V_100_ and V_150_ for the phantom cases, which revealed mean decreases of 10 cm^3^ and 8 cm^3^, respectively, when using ACE_uniform_ instead of TG-43.

Considering the corresponding control CBCTs, a very comparable magnitude and extent of dosimetric differences between TG-43 and ACE_uniform_ was observed. Statistical significance of these differences was proven for all examined parameters (*p* ≤ 0.03), except of $\mathrm{D}_{{2\mathrm{cm}^{3}}}$ bladder (*p* = 0.46), HR-CTV D_90_ (*p* = 0.99), V_100_ (*p* = 0.98), and V_150_ (*p* = 0.86) for cervix cases, PTV D_90_ (*p* = 0.08) for breast cases, as well as PTV D_90_ (*p* = 0.31) for the phantom cases. The maximum structure-related difference was obtained for ribs $\mathrm{D}_{{1\mathrm{cm}^{3}}}$ with an exposure of 200 ± 67 cGy (TG-43) and 195 ± 65 cGy (ACE_uniform_). The clinical relevance of the observed dosimetric deviations appeared therefore again to be limited.

**Fig. 6:**
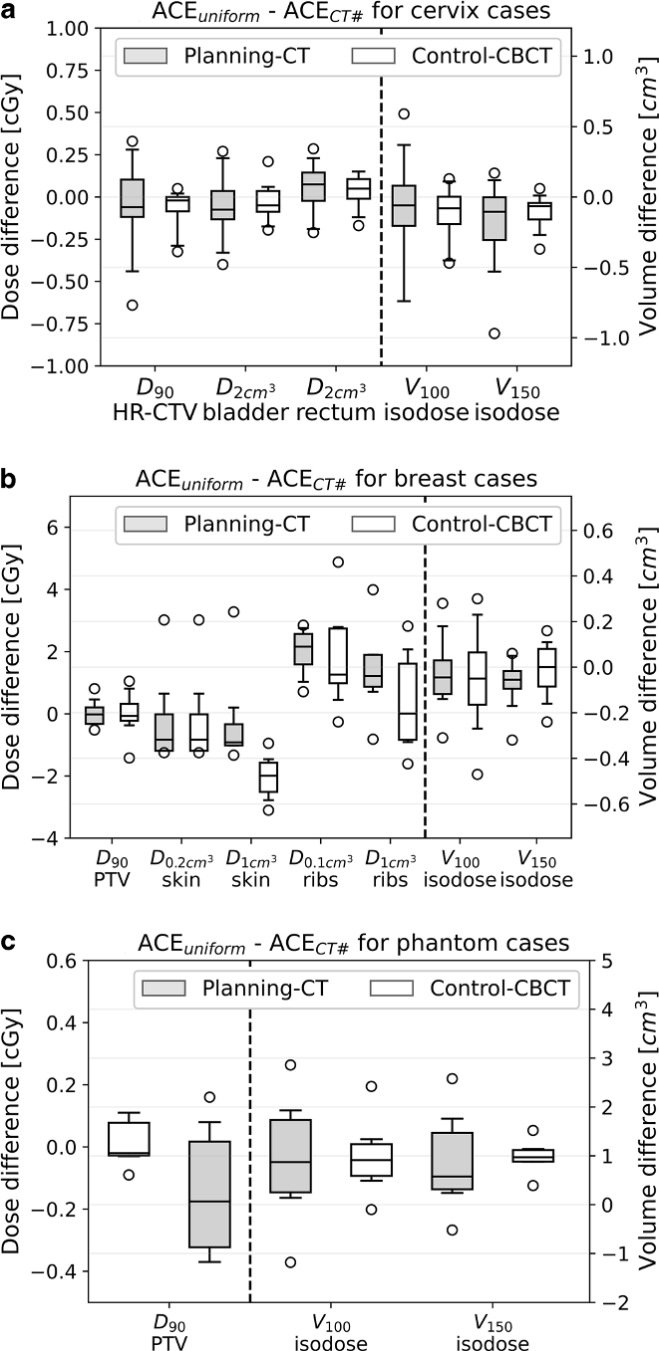
Dosimetric deviations (for single fractions) between ACE_uniform_ and ACE_CT#_ calculations performed on both planning CTs and control CBCTs for the examined cervix (**a**), breast (**b**), and phantom (**c**) cases

To investigate the impact of explicit considerations of CT numbers on the model-based dose calculation, we compared the differences between ACE_uniform_ and ACE_CT#_ obtained for both planning CTs and control CBCTs to each other. The corresponding results can be obtained from Table 1 and are visualized in Fig. 6 for each examined entity. Considering CT umbers instead of assuming only uniform materials did result in dosimetric deviations being most pronounced for structures located at tissue transitions and heterogeneities (e.g., at the lung–bone–soft tissue transition). Maximum averaged deviations in this respect were found for the ribs $\mathrm{D}_{{0.1\mathrm{cm}^{3}}}$ (change of 1.9 ± 0.8 cGy) for breast cases and V_100_ (change of 0.9 ± 1.4 cm^3^) for phantom cases on the planning CT. For the control CBCT scans, the maximum deviation between ACE_uniform_ and ACE_CT#_ observed for an individual patient amounted 4.9 cGy (ribs $\mathrm{D}_{{0.1\mathrm{cm}^{3}}}$) for a breast patient and single fraction. For cervix cases representing the examined entity located deepest inside the body, all observed structure-related dosimetric changes had an absolute magnitude < 0.7 cGy per fraction on both planning CT and control CBCT. The observed results, thus, suggested that taking into account CT numbers instead of assuming uniform materials for dose calculation did not lead to clinically meaningful deviations at all with respect to all entities examined in this work.

The comparison of the ACE_uniform_–ACE_CT#_ deviations between planning CTs and control CBCTs revealed no statistically significant difference (*p* > 0.18) for all examined dose parameters, with the exception of skin $\mathrm{D}_{{1\mathrm{cm}^{3}}}$ (*p* = 0.04) for breast patients. For the latter, mean dosimetric deviations of −0.2 ± 1.6 cGy and −2.0 ± 0.7 cGy per fraction were found considering the planning CT and control CBCT, respectively (Fig. 6b). This difference originated from the occurrence of slight beam hardening and scatter artifacts at the plastic buttons applied to avoid catheter slippage as mentioned in section ‘Patient workflow and anthropomorphic phantom study’ on the control CBCTs. Despite the statistical significance regarding this dose parameter, the reported differences between planning CT and control CBCT were once again considered to be of only limited clinical relevance for causing potential skin toxicity. In summary, between planning CT and control CBCT scans no relevant differences in model-based dose calculations considering CT numbers were found.

## Discussion

In the present work, we investigated model-based dose calculations using the Advanced Collapsed Cone Engine based on current state-of-the-art CBCT scans compared to calculations on conventional planning CTs. Examinations were performed for cervix and breast patients as well as for an anthropomorphic phantom equipped with superficial flaps. Except for skin $\mathrm{D}_{{1\mathrm{cm}^{3}}}$ (*p* = 0.04) for breast patients, we found no statistically significant differences (*p* > 0.18) in the ACE_uniform_–ACE_CT#_ deviations (i.e., in the model-based dose calculations assuming uniform materials as well as considering CT numbers) between CT and CBCT scans for all examined dose parameters. From our point of view, all of these differences even including skin $\mathrm{D}_{{1\mathrm{cm}^{3}}}$ appeared to be of limited clinical relevance, which has to be proven in future clinical outcome analyses. The TG-186 recommendation statement [24] “CBCT images should not be used for brachytherapy dose calculations when tissue heterogeneity information needs to be taken into account” thus seemed to be invalid using our modern state-of-the-art CBCT system and the examined MBDCA. The generalizability of our results to other CBCT systems and MBDCAs remains to be investigated in further studies.

Model-based dose calculation with ACE_CT#_ requires tissue electron densities as input parameters, which are obtained from the CT numbers of acquired CT or CBCT scans according to Eq. 1. To analyze potential CT number uncertainties for the CBCT scanner applied in this work, a phantom study was performed. For both examined phantoms, we found a reasonable agreement of the CT numbers and electron densities obtained from CBCT and conventional CT scans. This observation was concomitant with previously published results [28, 29], which also revealed a very good CT number accuracy of the ImagingRing for selected imaging protocols. To account for the slight differences found, a Hounsfield unit look-up table assigning CT numbers measured with a specific device to actual electron densities of examined materials/tissues is typically created for EBRT treatment planning [45]. For the Advanced Collapsed Cone Engine implemented in our brachytherapy TPS Oncentra Brachy, this assignment is defined by and fixed to Eq. 1, and no custom CT scanner specific density conversion table can be created as it would be feasible in other TPSs as, for example, RayStation. In general, verifying the agreement of the electron densities obtained by the CT and CBCT scans is considered important in technical CBCT scanner characterization regarding its applicability for model-based dose calculations. A corresponding QA of this agreement over time should also be added to corresponding QA routines of the ImagingRing, which have been published previously and revealed a good CT number stability over time [29].

With respect to the examined patient and anthropomorphic phantom cases, the evaluated dose parameters showed partly significant deviations between TG-43 and ACE_uniform_ dose calculations, regardless of whether considering planning CTs or control CBCTs. Taking into account tissue heterogeneities thus revealed a significant impact on resulting dose distributions. In addition, we observed that treatments of cervical cancer located deep inside the body and surrounded by soft tissue (i.e., approximating the water-equivalent environment assumed in the TG-43 formalism [21]) showed partly fewer dosimetric deviations dependent on the MBDCA than the breast and phantom cases with structures located closer at strong material/tissue heterogeneities. Our findings were in line with previous studies. For instance, Duque et al. [46] found that the TG-43 overestimates dose to liver tissue compared to TG-186 calculations in liver brachytherapy, with target coverage deviations as high as 1.5% of the total CTV volume and 3.5% of the prescribed dose. Zourari et al. [47] showed a TG-43-related dose overestimation for ribs and lung (on the order of 4% for the maximum point dose) as well as for skin (on the order of 6% for $\mathrm{D}_{{10\mathrm{cm}^{3}}}$) for accelerated partial breast brachytherapy. Scherf et al. [48] observed for mold-based skin brachytherapy a 6.4% lower PTV D_90_ using model-based dose calculations instead of TG-43. However, it has to be noted that the clinical relevance of the reported dose deviations still needs to be carefully evaluated for each considered entity, and further clinical investigations and dose–outcome analyses are aimed at in this respect to outline the clinical effects and benefits of model-based dose calculation prior to a potential routine application. In each case, we think that the choice of the dose calculation procedure definitely has to be consistent when conducting adaptive workflows by means of control CBCTs. This means that dose calculations on the control CBCT should be the performed just like the dose calculations during treatment planning were conducted, independent of whether TG-43 or ACE was chosen for this purpose. This becomes evident when considering the 10 cm^3^ decline in V_100_ between ACE_uniform_ and TG-43 obtained for the phantom cases, which would probably cause confusion in clinical routine if different dose calculation procedures on the planning CT and the control CBCT were used.

The explicit consideration of CT numbers in dose calculation (ACE_CT#_) instead of assuming uniform materials (ACE_uniform_) led to only small dosimetric variations in general. Hence, the change of TG-43 to ACE_uniform_ revealed a larger impact than additionally utilizing the CT numbers. All differences in the ACE_uniform_–CE_CT#_ variations between planning CT and control CBCT were out of statistical significance, with an exception for skin $\mathrm{D}_{{1\mathrm{cm}^{3}}}$ due to artifacts caused by the applied plastic buttons as reported in section ‘Comparison of dose calculation methods’. Apart from these visual artifacts, it has to be mentioned that the buttons also contain a high atomic number element for better visibility and high contrast in X‑ray imaging. Defining tissue types like “soft tissue” and “skin” as described in section ‘Comparison of dose calculation methods’ as well as the density conversion according to Eq. 1 may potentially not allow an exact dosimetric consideration of these objects. This has to be mentioned for completeness, although the corresponding dosimetric relevance is considered to be very low due to the small button size. Within the frame of this work, we therefore found that modern state-of-the-art CBCT scans can—in contrast to the TG-186 recommendation statement [24]—in principal be used for model-based dose calculations. We also showed that, in case of severe artifacts or CT number uncertainties occurring, performing model-based dose calculations assuming uniform materials with ACE_uniform_ does provide very similar results to ACE_CT#_ and can therefore represent a valuable alternative. The latter finding particularly implies that CBCT imaging can in principal always (no matter of how strong electron density uncertainties actually are) be used basis for a corresponding dosimetry, as long as material homogeneity is set in the TPS. Establishing smooth (re)planning workflows based on interventional in-room CBCT imaging on the brachytherapy ward [10, 28] combined with model-based dose calculations (without the need for patient transfer to distant CT scanners) is thus considered feasible in principle. However, please note that while we use the ImagingRing for adaptive quality assurance in our institution, we currently do not use it for definite initial treatment planning due to slight drawbacks in soft tissue differentiability compared to our high-end conventional CT scanner, which might hopefully be further reduced in the future by ongoing developments of this new machine.

Regarding implant variations during the brachytherapy course, we observed for both cervix and breast patients larger dosimetric deviations than caused by switching the dose calculation procedure. OARs as bladder and rectum affected by different filling states or rectal gas bubbles showed larger dosimetric variations than the actual target volume being fixed in position by the applicators. The same also holds for skin and ribs of breast patients. However, note that the dosimetric comparison between control CBCT and planning CT was based on a recontouring of OARs as well as a target volume transfer, which is a process associated with contouring uncertainties of potential dosimetric impact well described in literature [49–54]. For instance, Bell et al. [49] observed a $\mathrm{D}_{{2\mathrm{cm}^{3}}}$ change up to 3.6 Gy for OARs in MRI-guided gynecologic brachytherapy, where the image contrast is even improved compared to CT or CBCT. Due to the comprehensive literature already available in this respect, we did not analyze the impact of contouring uncertainties in the present work, which can be seen as a drawback. However, please note that the provided assessment of interfractional dosimetric changes served particular as an estimation allowing to put dose calculation differences between TG-43 and ACE into perspective. In this regard, we showed that the latter are small compared to the interfractional changes potentially occurring during the brachytherapy course. Although these findings represent a secondary result of this manuscript, they clearly suggested that performing an imaging-based treatment QA during brachytherapy is important. This also supports previous studies as for instance of Nesvacil et al. [55], which reported substantial dose variations and standard deviations for bladder and rectum $\mathrm{D}_{{2\mathrm{cm}^{3}}}$ of 0.6 ± 19.5% and 4.1 ± 21.7%, respectively, for cervix brachytherapy. For breast patients, a recent study observed geometric implant variations > 5 mm in up to 37% of all cases [8]. It has to be noted that performing a treatment QA cannot only help to assess requirements for treatment replanning, but also aims at the establishment of new workflows for improving overall implant stability. For example, we developed a decision-tree for assessing the need for treatment replanning for breast brachytherapy based on control CT images earlier, and found that 14% of all patients would have required treatment adaption [8]. Based on our findings, we implemented a breast positioning control workflow which substantially reduced $\mathrm{D}_{{0.2\mathrm{cm}^{3}}}$ skin as well as $\mathrm{D}_{{0.1\mathrm{cm}^{3}}}$ rib dose variations for the treated breast patients, and no patient of the examined cohort required treatment replanning anymore [6]. Based on this experience, we think that assessing the extents and causes of implant variations over time should be a considered measure to determine potential workflow improvements in each institution, and control CT/CBCT imaging can be a useful tool for performing dosimetric quality assurance to support this purpose.

Our study featured several limitations. At first, we performed dose calculations only based on the Advanced Collapsed Cone Engine implemented in Oncentra Brachy, but considered no other model-based dose calculations mentioned in the TG-186 recommendation [24] as well, as for instance Monte Carlo simulations. Second, our study was limited to a small patient number and larger cohorts would have been helpful to outline significance and relevance of the reported result even more. In addition, we did not consider further entities of interest as, e.g., head and neck patients, where the presence of strong heterogeneities between metal implants, air, bones, and soft tissue could impact dose calculations to a larger extent. This was, since corresponding CBCT-based treatment QA for these patients is not yet a component of our clinical workflow. For the same reason, we used an anthropomorphic phantom to evaluate dose calculations for patients suffering from superficial malignancies. Furthermore, we investigated only treatments performed with an ^192^Ir source, and no low-energy sources as typically applied for permanent brachytherapy [3] with dosimetric changes depending on CT number variations being expected to be larger [22]. Finally, we mainly focused solely on the physical dose differences and the corresponding suitability of CBCT imaging for model-based calculations. The assessment of dose–outcome and dose–toxicity relationships considering the differences in calculation procedures remains subject of future investigations, which are aimed at to evaluate and manifest actual clinical needs and requirements for dosimetry beyond TG-43 in brachytherapy. Nevertheless, we showed that CBCT imaging has the potential to support corresponding workflows in this respect.

## Conclusion

Model-based dose calculation based on modern CBCT imaging is considered suitable for cervix, breast, and skin brachytherapy and revealed only small dosimetric deviations compared to calculations on conventional CT scans. The TG-186 recommendation [24] “CBCT images should not be used for brachytherapy dose calculations when tissue heterogeneity information needs to be taken into account” appeared to be not valid using our CBCT device. However, partly significant differences between Advanced Collapsed Cone Engine calculations and the TG-43 formalism were found, implying the need for further clinical studies in this respect. Implant variations observed during the brachytherapy course led to dose variations larger than found between the various calculation procedures, revealing the importance of workflow-related treatment QA measures.

## Data Availability

The data that support the findings of this work are available from the corresponding author upon reasonable request.
